# Competition assays reveal a contribution of the C4 protein CCL-like domain to the fitness of tomato yellow leaf curl virus in the host plant

**DOI:** 10.1128/jvi.00900-26

**Published:** 2026-08-05

**Authors:** Man Gao, Chaonan Shi, Stephane Blanc, Rosa Lozano-Durán

**Affiliations:** 1 Department of Plant Biochemistry, Centre for Plant Molecular Biology (ZMBP), Eberhard Karls University, Tübingen, Germany; 2 PHIM, Université Montpellier, INRAE, IRD, CIRAD, Institut Agro 740764 , Montpellier, France; 3 Cluster of Excellence GreenRobust, Eberhard Karls University, Tübingen, Germany; Tsinghua University, Beijing, China

**Keywords:** symptoms, plant-virus interactions, geminiviruses, viral fitness, competition assays

## LETTER

Virus infections in plants frequently induce developmental alterations that manifest as disease symptoms. Despite the agricultural relevance of these virus-triggered changes, our understanding of their biological significance and of the molecular mechanisms underlying their appearance remains limited (1).

Recently, we reported that symptom development caused by the geminivirus tomato yellow leaf curl virus (TYLCV) in its natural host depends on a host-mimicking CCL-like domain present in the virus-encoded C4 protein. This domain mediates interaction with RCC1 domain-containing (RLD) proteins, which enables their sequestration at the plasma membrane and interferes with their functions in endomembrane trafficking and the establishment of cell polarity (1–3). A TYLCV mutant expressing a C4 protein carrying two amino acid substitutions in the CCL-like domain (TYLCV^C4^_CCLm3_), which impair interaction with RLDs, retained the ability to infect tomato plants but induced markedly attenuated symptoms. Comparison of virus accumulation in plants individually inoculated with TYLCV or TYLCV^C4^_CCLm3_ suggested that the mutant virus was not impaired in its ability to replicate and spread within the host. Interestingly, TYLCV-induced symptoms enhance attraction of its insect vector, the whitefly *Bemisia tabaci*, supporting the idea that symptom development may have evolved to promote viral transmission within this tripartite interaction (1, 4).

A limitation of our previous study, however, was that conclusions regarding within-host viral fitness were derived from individual infection experiments. While such assays provide valuable phenotypic information, they do not directly assess competitive performance. Within-host viral fitness refers to the relative ability of a virus genotype to replicate and spread within its host, and mixed-infection competition assays are considered the gold standard for its quantification because they directly measure the relative reproductive success of competing viral genotypes under identical environmental conditions (5).

**Fig 1:**
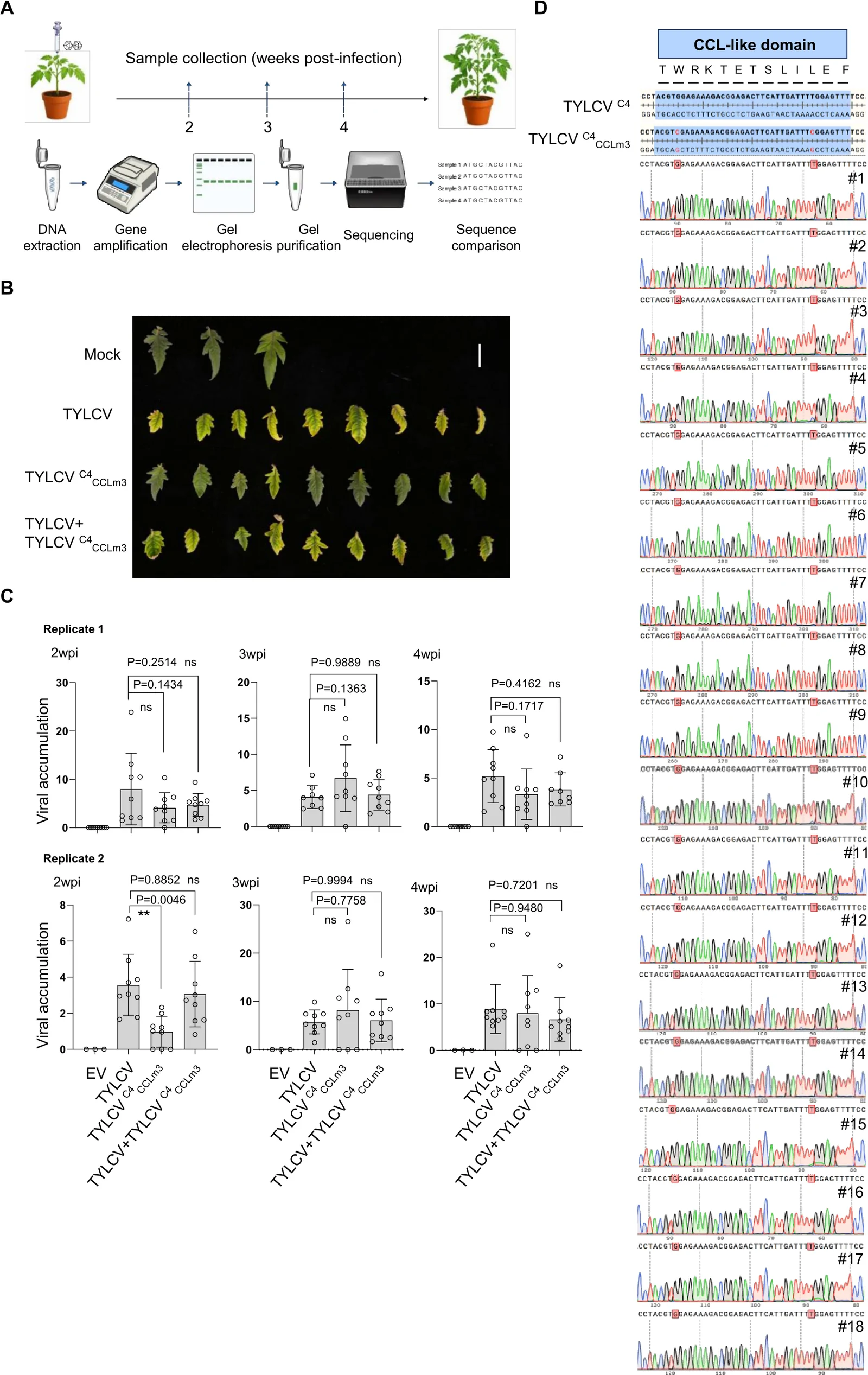
Consistent prevalence of wild-type TYLCV in mixed infections demonstrates a fitness penalty of the C4_CCLm3_ mutation. (**A**) Experimental design and workflow for the analysis of TYLCV + TYLCV^C4^_CCLm3_ mixed infections in tomato plants. (**B**) Symptoms caused by TYLCV, TYLCV^C4^_CCLm3_, or TYLCV + TYLCV^C4^_CCLm3_ co-infection in tomato leaflets; leaflets of mock-inoculated plants are shown for comparison. Images were taken at 4 weeks post-infection (wpi). Scale bar: 1.3 cm. (**C**) Virus accumulation in plants inoculated with TYLCV, TYLCV^C4^_CCLm3_, or co-inoculated with both (TYLCV + TYLCV^C4^_CCLm3_) at 2, 3, and 4 wpi. Results from two independent biological replicates are shown. Each dot represents an individual plant; error bars represent SD. Significant differences between groups were determined by one-way analysis of variance followed by multiple comparisons of means by applying a Tukey test; asterisks indicate statistically significant differences: **P < 0.01; ns, not significant, *P* ≥ 0.05. (**D**) Sequence alignments to the wild-type CCL domain-coding DNA region in the TYLCV C4 gene and corresponding Sanger chromatograms are shown for 18 co-inoculated (TYLCV + TYLCV^C4^_CCLm3_) tomato plants at 4 wpi. For each co-inoculated plant, a DNA fragment containing the CCL domain-coding region was PCR-amplified with primers tgacgttgtaccacgcatca and atgcctcgtttatttaaaatatatgcc, and the resulting fragment was gel-purified and Sanger-sequenced. Nucleotide positions mutated in TYLCV^C4^_CCLm3_ are in red squares. Peak colors correspond to individual nucleotides (A, T, C, and G).

To reassess the within-host fitness of TYLCV^C4^_CCLm3_, we performed competition assays through mixed infections with the wild-type virus. Two independent batches of nine tomato plants were inoculated with either TYLCV, TYLCV^C4^_CCLm3_, or both viruses simultaneously, and samples were collected at 2, 3, and 4 weeks post-inoculation (wpi) (Fig. 1A). Symptoms and viral accumulation were evaluated at each time point (Fig. 1B and C). Consistent with our previous findings (1), plants infected with TYLCV^C4^_CCLm3_ displayed significantly milder symptoms than plants infected with the wild-type virus, whereas viral accumulation was not consistently altered (Fig. 1C). Notably, symptoms observed in co-inoculated plants more closely resembled those induced by the wild-type virus (Fig. 1B).

To determine the relative abundance of wild-type and mutant viruses during mixed infection, we PCR-amplified the genomic region encompassing the CCL-like domain-coding sequence and analyzed the resulting amplicons by Sanger sequencing at all three time points. If the two viruses had equivalent within-host fitness, their relative signals in Sanger chromatograms might vary among individual plants, possibly because of genetic drift during plant colonization, but no consistent skew toward either sequence would be expected. Instead, the wild-type sequence predominated in nearly all analyzed plants, whereas the mutant sequence was detected only as faint traces in 2 of the 18 co-inoculated plants (#4 and #10) (Fig. 1D; Fig. S1). Temporal dynamics of genotype frequencies across individual samples are shown in Fig. S2 and reveal a gradual displacement of the mutant genotype that is evident already at 2 wpi. The consistent bias toward the wild-type genotype indicates that this outcompetes TYLCV^C4^_CCLm3_ during mixed infection, revealing a fitness cost associated with disruption of the CCL-like domain that had not been detected in single-infection experiments.

Our previous conclusions therefore require refinement. Although disruption of the CCL-like domain does not abolish infectivity, it compromises within-host viral fitness, resulting in displacement of the mutant virus during competition with the wild type. This reduction in fitness may contribute to the evolutionary maintenance of the CCL-like domain in plant hosts, with symptom development and enhanced insect-vector attraction arising as collateral consequences that ultimately favor viral spread. Whether the fitness defect associated with the C4^CCLm3^ mutation results directly from disruption of the interaction with RLD proteins or from an alternative molecular activity of C4 remains to be determined.

Collectively, this work not only provides new insight into the contribution of the CCL-like domain of TYLCV C4 to infection but also highlights the importance of utilizing competition assays for accurate assessment of viral fitness, since they can reveal biologically meaningful phenotypes that remain hidden in conventional single-infection analyses.

## SUPPLEMENTAL MATERIAL

Figures S1 and S2 jvi.00900-26-s0001.docx Sequence alignments and genotype frequencies.

ASM does not own the copyrights to Supplemental Material that may be linked to, or accessed through, an article. The authors have granted ASM a non-exclusive, world-wide license to publish the Supplemental Material files. Please contact the corresponding author directly for reuse.
