# Supplementary material for: Competition assays reveal a contribution of the C4 protein CCL-like domain to the fitness of tomato yellow leaf curl virus in the host plant
Source: J Virol. 2026 Aug 5;100(9):e00900-26. doi: 10.1128/jvi.00900-26 (PMC13595916; doi:10.1128/jvi.00900-26)
Supplement: Supplementary file 1 [file jvi.00900-26-s0001.docx]

**SUPPLEMENTARY MATERIAL**

**Competition assays reveal a contribution of the C4 protein CCL-like domain to the fitness of tomato yellow leaf curl virus in the host plant**

Man Gao^1^, Chaonan Shi^1^, Stephane Blanc^2^, Rosa Lozano-Durán^1,3^

^1^Department of Plant Biochemistry, Centre for Plant Molecular Biology (ZMBP), Eberhard Karls University, Tübingen, 72076, Germany. ^2^PHIM, Université Montpellier, INRAE, IRD, CIRAD, Institut Agro, Montpellier, France. ^3^Cluster of Excellence GreenRobust, Eberhard Karls University, Tübingen, 72076, Germany.

**Supplementary figure 1.**

**Supplementary figure 2.**

**Supplementary references.**

**Supplementary figure 1.** **Sequence alignments to the wild-type CCL domain-coding DNA region in the TYCLV C4 gene and corresponding Sanger chromatograms are shown for 18 co-inoculated (TYLCV + TYLCV ^C4^_CCLm3_ ) tomato plants at 2, 3 and 4 weeks post-inoculation (wpi).** For each co-inoculated plant, a DNA fragment containing the CCL domain-coding region was PCR-amplified with primers tgacgttgtaccacgcatca and atgcctcgtttatttaaaatatatgcc, and the resulting fragment was gel-purified and Sanger-sequenced. Nucleotide positions mutated in TYLCV ^C4^_CCLm3_ are in red squares. Peak colors correspond to individual nucleotides (A, T, C, and G).

**A**

**B**

**Supplementary figure 2.** **Temporal dynamics of genotype frequencies across individual samples.** Quantitative peak-ratio analysis of Sanger chromatograms displaying the relative abundance of the G155C (**A**) allele and T182C **(B**) (calculated as Peak Height_mutant_/(Peak Height_wt_+ Peak Height_mutant_) (Cao et al., 2011; Carr et al., 2009) across 18 unique, independent samples. Data points are plotted chronologically for each sample at 2 weeks post-infection (2 wpi, light green circles), 3 wpi (blue squares), and 4 wpi (red triangles) to illustrate the intra-sample longitudinal progression. Dashed vertical lines demarcate separate individual samples.

**REFERENCES**

**Cao, P., Wang, Q.J., Zhu, X.T., Zhou, H., Li, R., and Wang, W.P.** (2011). Quantitative determination of allele frequency in pooled DNA by using sequencing method. Journal of chromatography. B, Analytical technologies in the biomedical and life sciences **879**:527-532. 10.1016/j.jchromb.2011.01.014.

**Carr, I.M., Robinson, J.I., Dimitriou, R., Markham, A.F., Morgan, A.W., and Bonthron, D.T.** (2009). Inferring relative proportions of DNA variants from sequencing electropherograms. Bioinformatics **25**:3244-3250. 10.1093/bioinformatics/btp583 %J Bioinformatics.
